# Optimised, Broad NGS Panel for Inherited Eye Diseases to Diagnose 1000 Patients in Poland

**DOI:** 10.3390/biomedicines12061355

**Published:** 2024-06-18

**Authors:** Ewa Matczyńska, Marta Beć-Gajowniczek, Larysa Sivitskaya, Elżbieta Gregorczyk, Przemysław Łyszkiewicz, Robert Szymańczak, Maria Jędrzejowska, Edward Wylęgała, Maciej R. Krawczyński, Sławomir Teper, Anna Boguszewska-Chachulska

**Affiliations:** 1 Genomed S.A., 02-971 Warsaw, Poland; 2 Chair and Clinical Department of Ophthalmology, Faculty of Medical Sciences in Zabrze, Medical University of Silesia, 40-055 Katowice, Poland; 3 Chair and Department of Medical Genetics, Poznań University of Medical Sciences, 61-701 Poznań, Poland; 4 Centers for Medical Genetics Genesis, 60-529 Poznań, Poland

**Keywords:** inherited retinal diseases, retinal dystrophy, inherited optic neuropathy, multi-gene NGS IRD panel, multi-gene NGS inherited optic neuropathy panel, Central and Eastern Europe

## Abstract

Advances in gene therapy and genome editing give hope that new treatments will soon be available for inherited eye diseases that together affect a significant proportion of the adult population. New solutions are needed to make genetic diagnosis fast and affordable. This is the first study of such a large group of patients with inherited retinal dystrophies (IRD) and inherited optic neuropathies (ION) in the Polish population. It is based on four years of diagnostic analysis using a broad, targeted NGS approach. The results include the most common pathogenic variants, as well as 91 novel causative variants, including frameshifts in the cumbersome *RPGR* ORF15 region. The high frequency of the *ABCA4* complex haplotype p.(Leu541Pro;Ala1038Val) was confirmed. Additionally, a deletion of exons 22–24 in *USH2A*, probably specific to the Polish population, was uncovered as the most frequent copy number variation. The diagnostic yield of the broad NGS panel reached 64.3% and is comparable to the results reported for genetic studies of IRD and ION performed for other populations with more extensive WES or WGS methods. A combined approach to identify genetic causes of all known diseases manifesting in the posterior eye segment appears to be the optimal choice given the currently available treatment options and advanced clinical trials.

## 1. Introduction

Inherited retinal dystrophies (IRD) and inherited optic neuropathies (ION) are among the most common genetically determined ophthalmic diseases. Inherited retinal dystrophies represent the major group of hereditary eye diseases, affecting 1 in 1500 people. Their incidence may be even higher, as some dystrophies with mild, late-onset symptoms may remain undiagnosed and be attributed to ageing or recent inflammation.

They represent a very heterogeneous group of diseases and syndromes (at least 50 major types; for some diseases, symptoms are due to variants in one of many causative genes), in some cases with additional, extra-ocular symptoms (syndromic retinal dystrophies) [1].

Most retinal dystrophies are of autosomal recessive inheritance. However, there are some genes in which both recessive and dominant inheritance of dystrophies are possible, depending on the variants concerned. These dystrophy types may differ in their clinical manifestations, making the diagnosis of IRD more complex. The X-linked inheritance is relatively frequent, as presented by recent studies [2].

Some IRDs are due to variants in more than one gene (digenic inheritance). The involvement of the mitochondrial genome in IRD development should not be disregarded and its analysis should be included in order to identify causative variants for syndromic retinal dystrophies.

Inherited optic neuropathies (IONs) represent another heterogeneous group of inherited eye diseases, leading to vision loss due to the progressive death of retinal ganglion cells and, eventually, optic nerve atrophy, affecting around 1 in 10,000 people [3,4]. The most common IONs are caused by mitochondrial dysfunctions due to mutations in mitochondrial genes (Leber hereditary optic neuropathy, LHON) or in autosomal genes regulating mitochondrial functions (autosomal dominant optic atrophy, ADOA). A new form of LHON (LHONAR) due to biallelic mutations in the *DNAJC30* gene (whose product is needed for mitochondrial complex I repair) was described and may account for a large proportion of unsolved LHON cases [5].

Owing to the advances in the application of NGS methods in genetic diagnostics and a relative decrease in the cost of such analyses, several vast studies using multi-gene panel, WES, and WGS approaches have been published, increasing our knowledge and our ability to correctly identify the genetic background of IRD or ION [4,6,7,8]. Over 300 genes have been identified as having mutations that could cause retinal diseases and over 30 with inherited optic neuropathies [1,9,10].

For many years, genetic counselling in ophthalmology was based on electrophysiological tests and fundus imaging. In the last decade, fundus autofluorescence examination has become one of the basic tools for examining retinal dystrophies. Wide-angle fundus cameras and devices using adaptive optics have also appeared on the market and are considered useful in this group of patients. However, regardless of the imaging and functional tests performed, the phenotype usually does not allow us to specify which gene is affected, much less the mutation itself. The common diagnoses such as “retinitis pigmentosa” or “bull’s eye” maculopathy are very general and do not allow determination of the mutation, gene, type of inheritance, prognosis, and therapy options.

Eye diseases that begin with decreased visual acuity without detectable changes in the eye fundus pose a diagnostic challenge, and children with such symptoms are often accused of simulating visual deterioration. Performing electrophysiological tests, especially electroretinography, in young children is difficult and carries the risk of artefacts and low reliability.

As long as the price of genetic testing was high and there were no available therapies, it was often abandoned. The situation changed with the registration of the first gene therapy in ophthalmology. Voretigene neparvovec (Luxturna, Novartis) is an AAV2 vector containing human *RPE65* cDNA with a modified Kozak sequence, which raised hope among people with retinal dystrophies [11]. However, mutations of the *RPE65* gene, resulting in the phenotype of retinitis pigmentosa (RP) or Leber’s congenital amaurosis (LCA), constitute a small percentage of dystrophies and the effectiveness of treatment is often disappointing. This is probably due to many factors—therapy undertaken too late, when retinal cells are irreversibly damaged; toxic effects of mutated gene products; inflammation caused by viral vectors; and retinal changes secondary to vitrectomy (surgical procedure required to introduce the gene), e.g., macular hole.

However, due to the high incidence of congenital retinal dystrophies and need for a cure, other gene therapies are currently at various clinical phases, including another gene therapy for RP/LCA, a combination of gene therapy and an electronic device (so-called optogenetics) [12], as well as treatment for the most common X-linked RP type, due to mutations in the *RPGR* gene that reached phase III [13].

In the case of LHON or LHONAR, therapeutic options include idebenone, a short-chain benzoquinone, an antioxidant [14], and a future gene therapy for *MT-ND4* with Lumevoq [15]. Among successful clinical trials, there is phase I/II for Leber congenital amaurosis type 10 (LCA10) caused by *CEP290* mutations for intravitreal antisense oligonucleotide [16], while Stargardt disease and other *ABCA4*-linked retinopathies are the focus of the phase I/II study for an in vivo RNA exon editor delivered through a single vector [17].

Other advanced and positive gene therapy clinical trials include those in patients with LCA type 1 caused by mutations in *GUCY2D* [18] and the treatment of LCA5 gene-dependent LCA (type 5) [19]. A so-called “molecular photoswitch”, conferring light-sensing capabilities to special types of retinal neuron cells, could potentially become a new treatment in RP and choroideremia [20]. Another future treatment will probably rely on CRISPR-Cas9-mediated genome editing in human induced pluripotent stem cells derived from patients and subsequent subretinal transplantation of differentiated retinal cells. Successful results in cell cultures were achieved for several types of retinal dystrophies such as Stargardt disease [21] and LCA [22].

Results of several studies for groups or small cohorts of Polish IRD patients were published, including those for Stargardt disease [23,24] and RP [25,26,27]. Those studies used mostly targeted approaches such as Sanger sequencing of selected genes [27], single-nucleotide polymorphism microarrays or small NGS panels (in Leber congenital amaurosis [28,29]; in CRD [30]) or more comprehensive NGS panels (108 genes, [23,24]), while, in several studies, the WES approach was applied [25,26,30,31]. Sanger sequencing and SNP arrays, as well as an NGS panel, were used to search for the molecular diagnosis in a large group of patients with LHON/LHONAR [32]. 

A large cohort analysis was carried out for another multifactorial retinal degeneration disease—age-related macular degeneration (AMD) [33].

The aim of this paper is to summarise the results of almost four years of applying a multi-gene diagnostic NGS panel in one clinical diagnostic laboratory to analyse a large group of Polish IRD and ION patients from the entire country. The NGS panel approach was developed as a follow-up and a consequence of a previous WES-based study performed in the same centre [31]. 

This study represents the largest analysis performed for the Polish population to date and provides important insights into the molecular epidemiology of inherited eye diseases in Poland. The main objectives were to study indications, advantages, and disadvantages of using a multi-gene NGS panel in the examination of patients with retinal dystrophies and inherited optic neuropathies.

## 2. Materials and Methods

This study involved a cohort of 1005 patients with clinical symptoms of retinal dystrophies (889) or optic neuropathies (116) from several centres, including the Chair and Clinical Department of Ophthalmology at the Medical University of Silesia, Centers for Medical Genetics Genesis in Poznań, and the counselling unit of Genomed S.A. in Warsaw (Table S1). The subjects were included in the cohort between January 2020 and December 2023.

This study was approved by the Ethics Committee of Medical University of Silesia (Resolution No KNW/0022/KB1/105/13) and adhered to the tenets of the Declaration of Helsinki. Informed written consents were obtained from the participants. 

Diagnostic genetic testing was performed in the laboratory of Genomed S.A., Warsaw, Poland. Genomic DNA was extracted from the peripheral blood of each patient using a total DNA isolation kit (MagCore Genomic DNA Whole Blood Kit For Genotyping, RBC Bioscience Corp., New Taipei City, Taiwan), according to the manufacturer’s protocol, and subsequently quantified using the Qubit Fluorometer (Qubit dsDNA Quantification Assay Kits, Thermo Fisher Scientific, Dreieich, Germany). Genomic DNA derived from each sample was used as the input for constructing a custom multi-gene enrichment panel NGS library. Three versions of the custom NGS panel were used during the study as the target of the panel was expanded on an iterative basis.

The initial version of the NGS panel was designed to capture coding exons of 274 IRD and ION genes along with 20 bp flanking regions (File S1), including additional deep intronic ABCA4 variants. The panel was developed using the Roche NimbleGen SeqCap EZ method (Roche NimbleGen Inc., Madison, WI, USA). The NGS libraries for 337 patients were prepared according to the Roche NimbleGen SeqCap EZ protocol with KAPA HyperPlus Library Preparation Kit, starting with the input of 100 ng of genomic DNA for each sample [34]. 

Subsequently, the second and, finally, the third version of the panel were developed using Twist Bioscience custom panel approach (TWIST Bioscience, San Francisco, CA, USA), capturing coding regions of 331 and 375 IRD/ION genes (File S1), along with selected Clinvar pathogenic or likely pathogenic deep intronic variants for the targeted gene list and the mitochondrial genome spike-in. Libraries were prepared according to the Twist Target Enrichment Protocols, starting from 50 ng of genomic DNA per sample [35,36,37] for 271 and 397 patients using the second and third version, respectively. 

All libraries were sequenced on the NextSeq550Dx or NovaSeq6000 platforms (Illumina Inc., San Diego, CA, USA) in the PE150 mode, aiming at an average target coverage above 200×.

Initial processing of BCL files, along with demultiplexing, was prepared with the Illumina bcl2fastq. Raw FASTQ files were trimmed from adapter sequences and low-quality bases using Cutadapt 2.8 [38], followed by read mapping to GRCh38 (hg38) reference genome using Burrows–Wheeler Alignment tool [39] in the alt-aware mode. Duplicate read pairs were removed using MarkDuplicates from Picard tools package [40]. Alignment files were further processed according to the GATK best practice pipeline [41] using Haplotype Caller for variant identification. Subsequently, the quality metrics for the alignment and variants were examined, including an average coverage > 150× and the fraction of the targeted region covered at least 20× >99%. All variants within the targeted regions, above the default variant quality threshold, were annotated with Ensembl Variant Effect Predictor [42] and additional, internal variant databases, including POLGENOM [43]. Annotated variants were subjected to detailed variant analysis and interpretation in the in-house variant browser BroVar, using the ACMG/ACGS variant classification guidelines [44,45]. No prior automatic variant filtering was performed. Each rare variant (MAF < 0.01 for gnomAD v2.1.1 and gnomAD v3, with the exception of known pathogenic or hypomorphic more frequent variants) was viewed and assessed for pathogenicity with the given protein effect, variant frequency compared to the expected frequency for the considered disease, presence in known pathogenic variant databases such as Clinvar [46], HGMD Professional [47], and LOVD [48], using multiple in silico predictors, and taking into account previous knowledge about the particular variant in the in-house database. Segregation was assessed when possible. Pathogenic and likely pathogenic variants were confirmed using Sanger sequencing. Copy number variants were identified initially with CoNVaDING [49] and further CNV analysis was performed with GermlineCNVCaller from GATKv4 package [50]. Identified CNV variants were annotated using AnnotSV [51], followed by a detailed diagnostic interpretation, with a verification of their presence in the variant databases, such as ClinVar, HGMD Professional, LOVD, and disease-specific databases. Selected detected copy number variants were confirmed using the MLPA method. 

A patient case was considered solved even if one of the confirmatory variants for an autosomal recessive disease was classified as an uncertain significance variant with strong supporting evidence (hot VUS) and genotyping results were consistent with clinical data. If such variants were the only variants identified, a case was considered likely solved. 

## 3. Results

### 3.1. Cohort Specification

A total of 1005 patients underwent genetic testing using the custom target enrichment panel approach. The group included 467 (46.4%) women and 539 (53.6%) men. The majority of the cases were tested as proband only. However, the group included seven families, where both siblings or a parent–child duo underwent testing (Table S1). Patients were subjected to a detailed ophthalmological examination involving visual acuity testing, colour vision testing, autorefractometry, tonometry, perimetry, and dilated fundus examinations, including fundus autofluorescence, OCT, fluorescein angiography, and ERG/EOG depending on the phenotype. The type of examinations for a particular patient was chosen individually by the referring ophthalmologist. 

Some patients included in the cohort had undergone earlier genetic testing that was inconclusive, mostly due to its narrow scope. Additionally, eight patients had been previously tested using an early version of WES enrichment in the framework of the Strategmed project without a positive result [52].

Patients were divided into several phenotypic groups based on the given indications. For retinal dystrophy, the groups included were as follows: retinitis pigmentosa (RP) (233, 23.2%), Stargardt disease (155, 15.4%), cone–rod dystrophy (CRD) (76, 7.6%), Leber congenital amaurosis (LCA) (46, 4.6%), Usher syndrome (Usher) (46, 4.6%), achromatopsia (ACHM) (32, 3.2%), atypical retinitis pigmentosa (Atypical RP) (30, 3.0%), and congenital stationary night blindness (CSNB) (15, 1.5%). The last IRD group, denoted as Other RD, constituted an aggregate category for the remaining dystrophy types indicated, including Bardet-Biedl syndrome, choroideremia, macular dystrophies, Cohen syndrome, retinoschisis, Stickler syndrome, Aland Island eye disease, and Wagner syndrome. The subsequent two groups consisted of patients with an indication of ION: optic atrophy (88, 8.8%) and Leber hereditary optic neuropathy (LHON) (28, 2.8%). The LHON/LHONAR group was limited mostly to patients with heterogeneous clinical traits or where initial testing for primary LHON-causing mutations was negative. In case of no clear indication of retinal or ION disease type, the patient was assigned to the Unspecific group. When the indication showed an alternative of more than one type of disorder, the patient was assigned to the first type of disease listed. The initial patient phenotype group assignment was not changed even if the genetic testing results indicated a different type of IRD or ION.

Among the defined phenotypic groups, the predominance of men was particularly noticeable in LHON/LHONAR (71.4% vs. 28.6%), retinitis pigmentosa (57.1% vs. 42.9%), and in the aggregate category denoted as Other RD (59.3% vs. 40.8%), suggesting that these groups contained a substantial proportion of X-linked inherited disorders or diseases in which male patients present more severe clinical manifestations (Table S1, Figure S1).

The mean age at the time of referral was 28 (SD = 18), while three among the phenotypic groups, LCA, ACHM, and CSNB, manifesting early-infantile nystagmus, showed a notably lower mean age (Table S1, Figure S2). The most numerous were patients with the initial diagnosis of retinitis pigmentosa (23.2%, N = 233) and Stargardt disease (15.4%, N = 155) (Table S1). A relatively large group included patients with no clear indication of a particular type of retinal dystrophy, denoted as Unspecific (10.3%, N = 104).

### 3.2. Molecular Diagnosis—Success Rate

**Figure 1:**
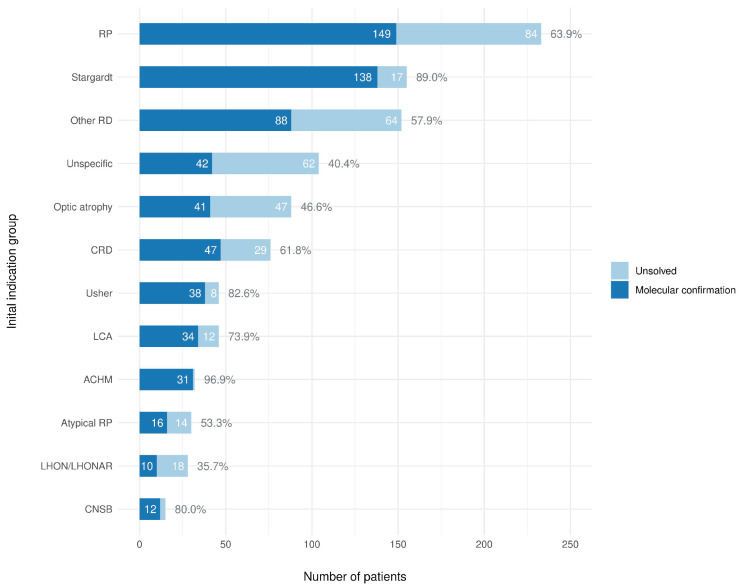
Diagnostic yield stratified into initial indication groups. Percentage of positive cases in each group shown on the right.

The definitive molecular diagnosis was reached in 646 (64.3%) cases (Figure 1, Table S2), with 595 positive cases among IRD groups (66.9%) and 51 positive cases in two ION groups (44.0%). The ACHM, Stargardt, and Usher syndrome phenotypic groups presented the highest diagnostic yield, 96.9%, 89.0%, and 82.6%, respectively. Conversely, 40.4% of patients with no clear IRD or ION type in their initial indication (Unspecific group) received a positive diagnosis, while, for the LHON/LHONAR indication, a molecular diagnosis was achieved only in 35.7% of cases (10/28).

A total of 113 (11.2%) patients had only one pathogenic variant associated with AR disease identified, most frequently in the *ABCA4*, *USH2A*, and *CDHR1* genes. In 157 (15.6%) patients, only VUS variants were reported, of which 15 were identified as most likely to be responsible for the patient phenotype, but additional information was required to reclassify those variants from hot VUS to likely pathogenic according to the ACMG/ACGS criteria [44,45], which was not available to the laboratory at the time of testing (Table S3). The remaining 90 (9.0%) patients received a result with no variants indicated.

### 3.3. Causative Genes and Variants

**Figure 2:**
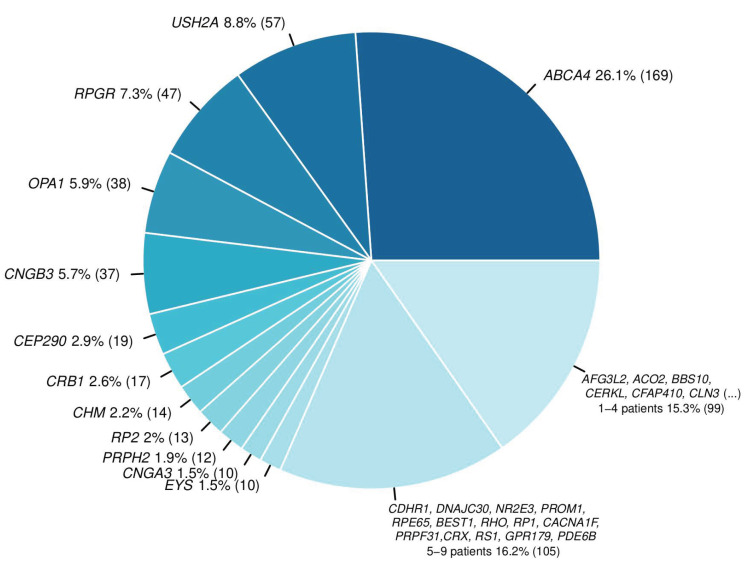
Distribution of genes with confirmatory variants among 646 distinct solved patients (total number of patients with confirmatory variants in denoted gene in brackets). The last two categories represent two sets of genes in which confirmatory variants were identified in 5–9 patients and 1–4 patients, respectively.

Confirmatory variants were found in 90 distinct genes. The largest cohort group consisted of patients with causative variants in the *ABCA4* gene (169, 26.1%), followed by *USH2A* (57, 8.8%) and *RPGR* (47, 7.3%) (Figure 2). A total of 41 (6.3%) patients presented confirmatory variants in genes that occurred only once in our cohort.

Overall, 521 distinct confirmatory variants were detected in the solved cases group. The majority of these were single-nucleotide variations (362, 69.5%), followed by 135 indels (25.9%), 111 small deletions and 24 small insertions. Copy number variations were represented by 24 different changes (4.6%). Most of the confirmatory variants were private, i.e., present in only one patient in the entire cohort (406, 77.9%). Subsequently, 91 (17.5%) variants were present in two to four solved patients. The remaining, most frequent 24 variants (4.6%), identified in at least five solved patients, are described in Table 1.

**Figure 3:**
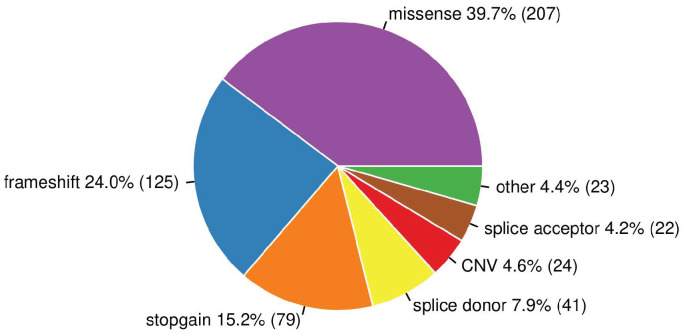
Distribution of 521 distinct causative variants into variant effect categories.

Looking at the effects of SNV, small insertions and small deletions across the set of distinct variants, the largest group represented missense variants (207, 39.7%), followed by frameshifts (125, 24.0%) and stop-gain variants (79, 15.2%) (Figure 3). Splicing variants accounted for 63 (12.1%) confirmatory variants. Only four variants (0.8%) with a synonymous effect were present in the set of distinct confirmatory variants, all with known or highly probable effects on splicing (Table S2). Eventually, two start-loss variants (0.2%) were identified in two different genes (Table S2).

**Figure 4:**
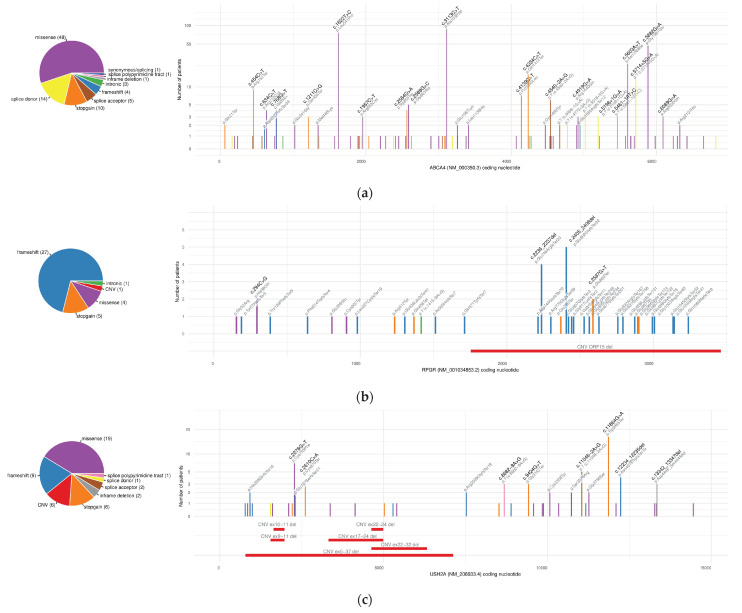
Distribution of causative variants along the transcript for three most prevalent causative genes in the cohort: (**a**) *ABCA4*, (**b**) *RPGR*, and (**c**) *USH2A*. The *x*-axis represents the transcript-coding nucleotide; the number of solved patients with the variant observed is denoted on the *y*-axis; colours of the variant bars represent the variant effect according to the respective pie plot on the left.

The *ABCA4* (NM_000350.3, NP_000341.2) c.3113C>T (p.Ala1038Val) and c.1622T>C (p.Leu541Pro) variants were the most prevalent confirmatory variants, observed in 87 patients each (Table 1 and Table S2, Figure 4). Both variants were identified within a complex haplotype p.(Leu541Pro;Ala1038Val) in 85 cases (15 times as a homozygote). Each of the variants was also present independently in two additional cases. The p.(Leu541Pro;Ala1038Val) haplotype allele frequency among the confirmatory variants in the cohort was estimated to be 30.7%, in line with previous observations for the Polish population [53] (File S1). The following variants were *ABCA4* c.5882G>A p.Gly1961Glu, identified in 47 positive cases (all in compound heterozygote), and hypomorphic c.5603A>T p.Asn1868Ile, identified in 24 positive cases (in trans with a loss-of-function allele). The hypomorphic c.5603A>T p.Asn1868Ile variant was also present in 73 positive cases with confirmatory variants in other genes than *ABCA4*, which confirms its very high incidence in the European population.

Both of the most prevalent confirmatory variants in *CNGB3* (NM_019098.5 and NP_061971.3) were frameshift variants (Table 1). The c.1148del p.Thr383IlefsTer13 variant, which is the most common pathogenic variant underlying achromatopsia, was found in 22 cases (in 7 cases as a homozygote). The second most prevalent variant in *CNGB3*, c.819_826del p.Arg274ValfsTer13, was found as confirmatory in 20 patients, only once as a homozygote. The most recurrent stop-gain variant, as well as the most frequent variant in *USH2A* (NM_206933.4, NP_996816.3), was c.11864G>A p.Trp3955Ter, found in 19 cases (3 homozygous cases). Interestingly, the second most prevalent variant in *USH2A* was a copy number variant c.(4627+1_4628−1)_(4987+1_4988−1)del (deletion of exons 22–24), which was identified in 14 cases, two times in homozygosity (Figure 4). The most recurrent deep intronic variant was *CEP290* (NM_025114.4, NP_079390.3) c.2991+1655A>G, identified in 14 patients, of which 5 were homozygous.

The total number of patients with a causal homozygous variant in the context of AR inherited disease was 116, accounting for 25.5% of all positive patients diagnosed with AR disease. The most frequent variant appearing as a homozygote was, as expected, the *ABCA4* haplotype p.(Leu541Pro;Ala1038Val), followed by *DNAJC30* (NM_032317.3, NP_115693.2) c.152A>G p.Tyr51Cys, observed as a homozygote in eight cases. Interestingly, the homozygous cases for the above-mentioned *DNAJC30* variant represented almost all positive cases with causal variants in *DNAJC30* (8/9), with one exception of a compound heterozygote in that gene.

**Figure 5:**
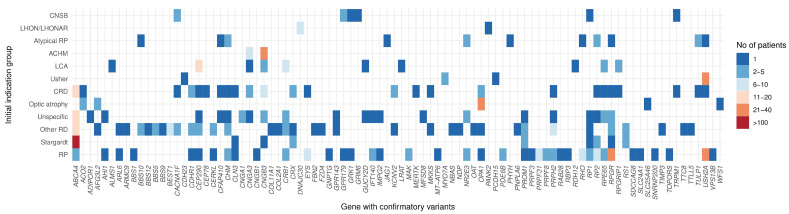
Distribution of all 646 solved patients into “confirmatory gene—initial indication group” pairs.

Looking at the diversity of genes in which confirmation of the diagnosis was found for initial indication groups (Figure 5), the RP group was the most diverse, with causative variants found in 40 different genes. The next group was the aggregate category, denoted as Other RD, with 36 genes harbouring the causative variants. This was expected since this category consists of various clinical phenotypes. The third group was CRD, with 25 different genes, followed by the Unspecific group, where the molecular diagnosis was found in 22 different genes. The least heterogeneous groups in terms of gene diversity were LHON/LHONAR and, as expected, ACHM with two different genes in each group. Interestingly, among the two genes in which pathogenic variants were identified for LHON/LHONAR, in addition to *DNAJC30*, the *PANK2* gene was found. *RPGR* was uncovered as the gene with confirmatory variants in the largest number of initial indication groups (CRD, RP, atypical RP, Other RD, Unspecific, and Stargardt), which may suggest a high clinical variability in phenotypes for patients with pathogenic variants in this gene.

### 3.4. Deep Intronic Variants

The systematic update of the panel target region with known pathogenic or likely pathogenic deep intronic variants (more than 20 bp from exon–intron boundary), based on the Clinvar database and the literature review, allowed for a molecular diagnosis in 19 cases. Most of these (14/19) were patients with a deep intron variant in *CEP290* (NM_025114.4:c.2991+1655A>G), as described above. In a further five cases, the deep intronic variants were unique to each patient. Three deep intronic causal variants were found in *ABCA4* (NM_000350.3:c.4253+43G>A, c.859−517G>A, and c.5196+1137G>A), all in *trans* with different pathogenic coding variants. The *ABCA4* NM_000350.3:c.859−517G>A variant was actually a novel deep intronic variant, found within a region where other known pathogenic deep intronic *ABCA4* variants have been observed. One deep intronic heterozygous variant was identified as causal in *OPA1* (NM_130837.3:c.610+360G>A). Finally, a deep intronic homozygous variant was found in *SDCCAG8* (NM_006642.5:c.740+356C>T) in a challenging low complexity sequence region. This was the only case in which the confirmatory variant was found in the *SDCCAG8* gene. All of the deep intronic variants described above can significantly contribute to the pathogenicity of inherited eye diseases by affecting splicing and thus gene expression.

### 3.5. Copy Number Variants

A total of 24 different copy number variants were detected as confirmatory changes in 44 positive patients (6.8%) (Table 2 and Table S2). In nine cases, the positive molecular diagnosis was possible solely due to the CNV analysis. The gene with the highest number of different CNV changes was *USH2A*, with six distinct changes, leading to a positive molecular diagnosis in 23 cases. Also, a higher number of unique changes was observed in the *EYS* and *RP2* genes, each harbouring three different CNVs, which enabled molecular diagnosis for four and three patients, respectively. In two cases, a deletion of all gene exons was identified (*CHM* and *RP2*). The majority of confirmatory CNV variants were deletions of two to three exons (22/24). Copy number duplications were reported as causal only for the *EYS* gene. A total of six single exon events were identified in eight cases (in *CNGB3*, *EYS*, *PRPH2*, *RP2,* and *RPGR*—spanning exon 15b—ORF15). The most frequent CNV change, and the only one observed as a homozygote, was the described above deletion of three exons in *USH2A* (ex 22–24del). The second most prevalent change was the deletion of exons 10–11, observed in five distinct patients, also in *USH2A*. The majority of distinct CNV changes (19/24, 79%) were observed only once throughout the cohort. It is worth noting that, for the *RAB28* gene, in which a causal homozygous CNV deletion of exons 5–7 was identified in one of the patients, there has been no information on pathogenic CNVs to date, according to the HGMD Professional database.

### 3.6. Novel Variants

A total of 91 possibly novel, SNV or small indel causal variants were uncovered in this study (Table S4). These variants have been selected as not reported in the NCBI Clinvar [46], HGMD Professional [47], NCBI dbSNP [54], gnomAD v2.1.1 [55], and Mastermind [56]. Five of these variants have been observed twice within the cohort. Three of them were found in individuals who do not appear to be related, but we cannot completely rule out this possibility; the remaining two variants were found in apparently related individuals.

The largest number of novel variants was detected in *ABCA4* (12, 13.2%). The second highest number of new variants was observed for the *RPGR* gene (9, 9.9%); six of them were localised within the most challenging *RPGR* ORF15 (Figure 4). Their detection was most likely due to improved coverage for this region, especially the purine-rich middle region. Surprisingly, the third gene with the highest number of novel variants identified was *OPA1* (8, 8.8%), although it actually ranked fourth according to the number of total confirmations (38, 5.9%) in the cohort. This may be due to the proportionally lower number of novel variants in *USH2A*, in which only five (5.6%) small novel variants were found (not including CNVs).

Interestingly, 40.7% (37/91) of novel variants were frameshifts, representing a noticeable increase over the percentage of frameshifts in the total set of causal variants (24.0%). The second most numerous group among the novel variants was missense variants (29.7%), followed by stop-gain variants (14.3%), splicing variants (13.2%), one deep intronic variant, and one inframe deletion.

### 3.7. Inheritance Patterns, Incomplete Penetrance

Considering solely the patients for whom a genetic diagnosis was achieved, autosomal recessive (AR) inheritance was prevalent and accounted for 69.6% (450/646) of solved cases. Autosomal dominant (AD) inheritance accounted for 16.6% (107/646) of cases with a genetic diagnosis, while X-chromosome-linked (XL) inheritance was demonstrated for 13.9% (90/646) of solved cases. Only in one patient (0.15%) was a known pathogenic mitochondrial DNA variant identified (NC_012920.1:m.9185T>C, rs199476138), with an 87% heteroplasmy in lymphocytes.

A relatively high proportion of patients carrying mutations in genes located on the X chromosome (13.88%) could be partially due to the improved analysis of the *RPGR* gene that was the most recurrently mutated X-linked gene (47 cases, 52.22%) in our cohort, significantly exceeding *CHM* (14 cases, 15.56%), *RP2* (13 cases, 13.83%), *CACNA1F* (7 cases, 7.78%), and *RS1* (6 cases, 6.67%), while only three cases were due to mutations in *GPR143* and *NDP*. The majority of X-linked variants identified were hemizygous (72/90, 80.0%) and confirmed clinical diagnosis in male patients, with only 18 female patients considered as solved cases. For those female RP patients, with a wide range of phenotype severity, only one heterozygous pathogenic variant was identified either in *RPGR* (eight cases) or in *CHM* and *RP2* (five cases each). In several cases (39/646, 6.0%), pathogenic, likely pathogenic variants, and hot VUSs were found in genes where IRD-associated variants can act either in a dominant or recessive manner (*ACO2*, *BEST1*, *GUCY2D*, *NR2E3*, *PROM1*, and *RP1*) and both manners of inheritance could be observed in our cohort according to the literature and database search.

Among the patients with an indication of optic neuropathy, where mutations in *DNAJC30* were identified, genetic diagnosis was available for eight male and only one female patient, confirming reports on its incomplete penetrance observed in women. There was only one patient with a double genetic diagnosis (a heterozygous mutation in *OPA1* and a homozygote in *OAT*, presented as a case report in Skorczyk-Werner et al., 2021 [57]), while some patients carried an additional monoallelic pathogenic or likely pathogenic variant or VUS in an IRD-associated gene.

## 4. Discussion

We have attempted a broad characterisation of variants causing inherited retinal dystrophies and inherited optic neuropathies in the Polish population. The results include data for more than 1000 patients analysed with a targeted IRD/ION-specific panel over a period of 4 years. The custom panel approach identified the molecular cause of the disease in 64.31% of patients. This result is comparable in terms of diagnostic yield with the results of other genetic studies of IRD in different populations [3,6,7,8]. Similarly, the diagnostic yield for the ION patients was comparable to the results obtained recently by Gilhooley et al. 2024 [4]. This let us suggest that a well-designed, frequently updated gene panel, which includes the analysis of known deep intronic variants, mitochondrial variants, and CNVs, is capable of producing results comparable to those obtained by much more costly and extensive WES and WGS methods.

This is the first study of such a large group of patients with inherited eye diseases in the Polish population. Our findings are in line with other studies of large European cohorts that included both syndromic and non-syndromic patients with the prevalence of IRD-associated mutations in the *ABCA4* and *USH2A* genes [3,6,58], followed by *RPGR*, similarly to newly performed studies [3,6]. In several studies where genetic testing was performed before the year 2020, the proportion of *RPGR* variants reported was significantly lower [58,59], confirming the importance of improvement of *RPGR* gene sequencing and bioinformatic analysis.

Our results support the conclusion by Ścieżynska et al. 2016 [53] of a high frequency of the p.(Leu541Pro;Ala1038Val) haplotype in the *ABCA4* gene. The allele frequency among the confirmatory variants in the cohort was estimated to be 30.7%, which is consistent with the result presented by Ścieżynska et al. 2016 [53] (File S1).

The most recurrent variant in *USH2A* (stop-gain c.11864G>A p.Trp3955Ter) was similarly demonstrated to be specific for the Eastern and Central Europe population [60], while the most frequent *DNAJC30* c.152A>G p.Tyr51Cys variant is a founder mutation widespread in Polish patients (nearly 95% of causative alleles), possibly with its origin in Poland or Ukraine [32].

Our study uncovers the high frequency of the copy number variant NM_206933.4:c.(4627+1_4628−1)_(4987+1_4988−1)del (deletion of exons 22–24 of *USH2A*) in the Polish population, identified in 14 apparently unrelated patients (2 homozygotes) and, therefore, the second most frequent *USH2A* variant in our cohort (Figure 4). Interestingly, this variant has been previously reported in single patients of European (in three cases—Slavic) origin [58,61,62,63,64] and, when homozygous, associated with a late-onset non-syndromic RP [62]. In our patients with the biallelic deletion of *USH2A* exons 22–24, late-onset RP with a partial deafness were reported, while, in the patients being compound heterozygotes in *USH2A,* more heterogeneous symptoms and earlier onset of RP and deafness were observed. A possibility of the founder effect for this CNV variant in the Polish population requires further investigation.

Our data on the frequency of the prevalent variants in the Polish population could be compared with data for two cohorts of a similar size and composition—representing unselected groups of inherited retinal disease patients from Italy (Karali et al., 2022 [6]) and IRD/ION patients from Germany (Weisschuh et al., 2023 [3]). The prevalence of pathogenic variants in Poland is more similar to that in Germany, with a similar set of five most frequent mutations (four in *ABCA4* and one in *CNGB3*). The complex haplotype in *ABCA4* p.(Leu541Pro;Ala1038Val), however, is the most frequent only in the Polish cohort, while the third *ABCA4* variant c.5882G>A p.Gly1961Glu is by far the most common in Italy and the second one in Germany. The hypomorphic *ABCA4* c.5603A>T p.Asn1868Ile variant—the most common in the German cohort and the fourth in the Polish group—seems underrepresented in the Italian cohort. This may reflect population biases, as it can be observed for other, less frequent variants, prevailing only in single populations. Founder effects could add, however, to the observed frequencies, as in the case of the *RHO* c.473C>A variant in the Italian cohort or the *USH2A* copy number variant (deletion of exons 22–24) in the Polish group.

In addition, expanding the scope of the study to include known deep intronic pathogenic variants allowed confirmation of the diagnosis in a further 19 cases, with confirmation in 7 cases based solely on deep intronic variants. Thus, some of the advantages of the WGS approach have been effectively captured by a more focused assay.

A comprehensive list of analysed genes allowed us to detect the possible cause of the disease for the female patient with a severe LHON-like phenotype. Finding *PANK2* variants as probable causative variants in this case would not be possible without the use of a wide NGS panel.

This study identifies novel CNV and SNV/indel variants, including 12 splicing variants, 1 intron variant (which may impact splicing), and 37 frameshift variants, notably 6 in *RPGR*. The high frequency of the frameshift (loss-of-function) variants in the *RPGR* gene, predominantly in ORF15, a mutational hotspot, leading to a premature translation termination, one of interesting results in this study, reflects the main mechanism of the pathogenicity in *RPGR*-related RP, already noted by other groups [13].

Two novel variants—one in *CRB1* (NM_201253.3:c.1342C>T) and in *DNAJC30* (NM_032317.3:c.293A>G)—have been recently published as a part of a description of a small group of patients with distinct clinical symptoms [29,32]. Additionally, our study allowed us to repeatedly identify rare variants (without any record in gnomAD v2.1.1 and ClinVar), apparently more frequent in the Polish population—in *RPGR* ORF15 (NM_001034853.2:c.3142_3143dup) [27,30] and in *EYS* (NM_001142800.2:c.1836_1837del) [24], uncovering some specific genetic traits of the Polish population.

### 4.1. Possible Reasons for Negative Results

We believe that there could be several reasons for negative results in our cohort. When considering patient selection, there is a possibility that not all of the patients were suffering from retinal dystrophies or optic neuropathies. Some patients were referred with nonspecific symptoms or as a part of a differential diagnosis. This observation would be supported by the low rate of positive results for the Unspecific phenotype patient group. A consultation at an expert ophthalmology centre followed by a genetic counselling session with a specialist in ophthalmogenetics could significantly increase the diagnostic success rate.

Furthermore, variants may have gone undetected by the bioinformatic pipeline we used due to difficulties in sequencing or mapping the area in question (e.g., extreme GC bias and low complexity sequences). We are aware that this situation may have occurred in the ORF15 exon of the *RPGR* gene in the first version of the panel, where coverage of the middle section of ORF15 was far from optimal, which was addressed in the second panel version. Additionally, due to the known problem of low-quality mapping in the *OPN1LW* and *OPN1MW* genes (caused by the high similarity of the sequences of those genes), some variants associated with blue-cone monochromatism may be missing. The results of CNV analysis in exons with high coverage variability across samples may have been suboptimal. Balanced structural variants also remain outside the detection scope of the panel due to the lack of coverage in the potential breakpoint regions; these types of variants are still best detected by the WGS approach. Although we have taken care to include known pathogenic deep intronic variants in the annual panel enrichment updates, it is possible that not all newly described variants are covered.

Mitochondrial genome analysis was added as a default option to the third version of the multi-gene panel; therefore, some mitochondrial variants could be missed for this cohort. Moreover, the bioinformatic analysis of structural variants in mtDNA was not included. Mitochondrial variants present solely in the retinal cells could also be theoretically missed as venous blood was the only source of DNA, although we are convinced that sensitivity of mtDNA sequencing and analysis allowed us to identify variants in heteroplasmy as low as 1%.

Additionally, the *DNAJC30* gene was not included in the first version of the NGS panel, which, along with the mitochondrial analysis issues mentioned above, may account for the low success rate for the limited LHON/LHONAR phenotypic group presented in this study.

### 4.2. Additional Testing Available after a Negative Result

Based on our results, including the findings for a limited group of patients previously tested using the WES approach, a regularly updated and optimised NGS panel is a better diagnostic option than WES, because it allows improved coverage in selected and cumbersome regions to be obtained and a comparable success rate [3,4,6,7,8], still at a lower cost and without unnecessary secondary findings that may be difficult to interpret. A custom version of an exome enrichment, including additional probes that would improve CNV identification and sequencing of deep intron variants, could be envisaged as a possible option with a similar diagnostic yield.

WGS analysis, however, according to some reports [3], could allow for up to 10% additional diagnoses when compared to WES and targeted approaches. This is a consequence of a more uniform coverage of all the analysed regions, the possibility of identification of an additional set of structural variants (translocations and inversions) due to the much better analysis of breakpoints, as well as a more comprehensive sequencing of variants in noncoding regions. Earlier reports [58] suggested that detection of even 2–3 exons’ deletions and improved coverage of GC-rich regions (such as the first exon of *GUCY2D*) are the major advantages of WGS, but our results show that this may be achieved using an optimised multi-gene panel. Both WES and WGS approaches, however, have the potential of identifying new causative genes for inherited eye diseases.

### 4.3. Therapeutic Options Arising

For nine patients with *RPE65* mutations (almost 1% of our cohort), there is already a gene therapy available, even if it is not fully efficient. Idebenone treatment seems a promising option for LHON/LHONAR patients not only with mtDNA but also with *DNAJC30* variants and the type of mutation has a prognostic significance [65]. For other patients with positive genetic results, involving pathogenic variants in most frequently mutated genes (such as *ABCA4* and *RPGR*), there will likely be new therapies available soon, with genome editing as the most plausible option. An antioxidant nanotherapeutic treatment could be another option, available even earlier [66]. It has been proposed for diseases affecting the posterior segment of the eye and it consists of polycaprolactone nanoparticles delivering antioxidative drugs to the retina with a long-lasting effect.

### 4.4. Diagnostic Algorithm Proposed for IRD and ION

We suggest that a diagnostic algorithm for IRD and ION would be as follows:

- Consultation at an expert ophthalmology centre, including all basic and advanced ophthalmologic measurements;
- Consultation at a genetic counselling unit with a specialist in ophthalmogenetics or with a clinical geneticist with experience in ophthalmogenetic and neurodegenerative diseases;
- NGS testing of the proband using a comprehensive ophthalmology panel (up to 400 genes), including mitochondrial genome, deep intronic variants, and CNV analysis;
- After-test genetic counselling;
- Familial testing.

In the case of negative results:

- Reanalysis of data after 2 years for carriers of VUSs being potential confirmatory variants;
- WES or, preferably, WGS only in unsolved cases of proven genetic origin;
- WGS advised mostly for carriers of pathogenic variants in genes responsible for AR diseases.

## 5. Conclusions

Our results confirm that at least two thirds of patients referred for genetic testing by ophthalmologists experienced in IRD and ION diagnosis can expect a positive test result based on a multi-gene panel whose price constantly decreases. In countries where reimbursement of genetic tests is limited, information about the test’s effectiveness is particularly important because it may encourage or discourage testing.

The panel used in this study includes all known diseases manifesting in the posterior segment of the eye—the retina, choroid, vitreous body, and optic nerve. In many cases, the phenotype is complex and involves changes in multiple structures of the posterior segment of the eye. Therefore, a combined approach to identify genetic causes of such a wide range of diseases seems to be the optimal choice.

The growing number of clinical trials in retinal diseases and possible prevention of the main cause of functional blindness—AMD, a disease largely genetically determined—will increase the number of tests performed. Our study is the first to cover such a broad spectrum of individuals afflicted with IRD and ION in this region of Europe. Moreover, our collected diagnostic data possess sufficient robustness to suggest or validate the occurrence of genetic variants and types of diseases specific for this region.

## Figures and Tables

**Table 1:** List of 24 most prevalent causative variants in the described cohort, observed in at least 5 solved cases.

Gene Symbol	Distinct Patients with Variant	Variant Class	HGVSc	HGVSp	Consequence	dbSNP	gnomAD_2.1.1 AF Total	Clinvar ID
*ABCA4*	87 *	SNV	NM_000350.3:c.3113C>T	NP_000341.2:p.Ala1038Val	missense	rs61751374	0.001754572	7894
*ABCA4*	87 *	SNV	NM_000350.3:c.1622T>C	NP_000341.2:p.Leu541Pro	missense	rs61751392	0.000162652	99067
*ABCA4*	47	SNV	NM_000350.3:c.5882G>A	NP_000341.2:p.Gly1961Glu	missense	rs1800553	0.004564289	7888
*ABCA4*	24	SNV	NM_000350.3:c.5603A>T	NP_000341.2:p.Asn1868Ile	missense	rs1801466	0.042191347	99390
*CNGB3*	22	small deletion	NM_019098.5:c.1148del	NP_061971.3:p.Thr383IlefsTer13	frameshift	rs397515360	0.001750039	5225
*CNGB3*	20	small deletion	NM_019098.5:c.819_826del	NP_061971.3:p.Arg274ValfsTer13	frameshift	rs775796581	6.01195 × 10^−5^	374027
*USH2A*	19	SNV	NM_206933.4:c.11864G>A	NP_996816.3:p.Trp3955Ter	stopgain	rs111033364	0.000116791	2357
*ABCA4*	16	SNV	NM_000350.3:c.4234C>T	NP_000341.2:p.Gln1412Ter	stopgain	rs61750137	7.0729 × 10^−6^	99263
*CEP290*	14	SNV	NM_025114.4:c.2991+1655A>G		intronic	rs281865192	0.000127755	1337
*USH2A*	14	CNV	NM_206933.4:c.(4627+1_4628−1)_(4987+1_4988−1)del		deletion of exons 22–24	-	0	179215
*ABCA4*	13	SNV	NM_000350.3:c.5714+5G>A		splice donor 5th base	rs61751407	0.000297145	99403
*CRB1*	13	SNV	NM_201253.3:c.2843G>A	NP_957705.1:p.Cys948Tyr	missense	rs62645748	0.000202696	39614
*ABCA4*	9	SNV	NM_000350.3:c.454C>T	NP_000341.2:p.Arg152Ter	stopgain	rs62646861	1.59253 × 10^−5^	99300
*DNAJC30*	9	SNV	NM_032317.3:c.152A>G	NP_115693.2:p.Tyr51Cys	missense	rs61732167	0.001248506	976691
*CDHR1*	8	SNV	NM_033100.4:c.783G>A	NP_149091.1:p.Pro261=	synonymous, splice region	rs147346345	0.003051605	301224
*ABCA4*	7	SNV	NM_000350.3:c.4139C>T	NP_000341.2:p.Pro1380Leu	missense	rs61750130	0.000233633	7904
*USH2A*	7	SNV	NM_206933.4:c.2276G>T	NP_996816.3:p.Cys759Phe	missense	rs80338902	0.000967694	2356
*ABCA4*	6	SNV	NM_000350.3:c.4540−2A>G		splice acceptor	rs61752435	3.9765 × 10^−6^	92869
*CNGB3*	6	SNV	NM_019098.5:c.1578+1G>A		splice donor	rs372006750	1.99893 × 10^−5^	189031
*ABCA4*	5	SNV	NM_000350.3:c.2588G>C	NP_000341.2:p.Gly863Ala	missense	rs76157638	0.004295095	7879
*CNGA3*	5	SNV	NM_001298.3:c.1641C>A	NP_001289.1:p.Phe547Leu	missense	rs104893617	0.000159178	9478
*GPR179*	5	small deletion	NM_001004334.4:c.984del	NP_001004334.3:p.Ser329LeufsTer4	frameshift	rs770066665	0.000536462	31204
*RPGR*	5	small deletion	NM_001034853.2:c.2405_2406del	NP_001030025.1:p.Glu802GlyfsTer32	frameshift	rs398122960	0	91389
*USH2A*	5	SNV	NM_206933.4:c.11048-2A>G		splice acceptor	rs200871041	3.9827 × 10^−6^	553421

*—identified in 85 cases within a complex haplotype p.(Leu541Pro;Ala1038Val), in two cases as independent variant.

**Table 2:** List of all 24 distinct causative CNV variants observed in solved cases.

Gene Symbol	Distinct Patients with Variant	Zygosity	Description	HGVSc	gnomAD_2.1.1 SVs	Clinvar ID
*BBS5*	1	HET	deletion of exons 1–2 in NM_152384.3	NM_152384.3:c.(?_−1)_(142+1_143−1)del	-	2443002 (Pathogenic)
*CHM*	1	HEMI	deletion of all exons in NM_000390.4	NM_000390.4:c.(?_−1)_(*1_?)del	-	-
*CLN3*	2	HET	deletion of exons 8–9 in NM_001042432.2 (1.02-kb deletion in CLN3)	NM_001042432.2:c.461−280_677+382del	0.000968 (DEL_16_153550)	3552 (Pathogenic)
*CNGB3*	2	HET	deletion of exon 15 in NM_019098.5	NM_019098.5:c.(1662+1_1663−1)_(1781+1_1782−1)del	-	427720 (Pathogenic)
*EYS*	2	HET	deletion of exon 32 in NM_001142800.2	NM_001142800.2:c.(6424+1_6425−1)_(6571+1_6572−1)del	-	565297 (Pathogenic)
*EYS*	1	HET	duplication of exons 27–29 in NM_001142800.2	NM_001142800.2:c.(4790_5836−1295)_(6002_6172)dup	-	-
*EYS*	1	HET	duplication of exon 22 in NM_001142800.2	NM_001142800.2:c.(3243+1_3244−1)_(3443+1_3444−1)dup	-	565296 (Pathogenic)
*KCNV2*	1	HET	deletion of exons 1–2 in NM_133497.4	NM_133497.4:c.(?_−1)_(*1_?)del	0.00009219 (DEL_9_98700)	59054 (VUS)
*PRPF31*	1	HET	deletion of exons 4–5 in NM_015629.4	NM_015629.4:c.(238+1_239−1)_(420+1_421−1)del	-	-
*PRPF31*	1	HET	deletion of exons 5–7 in NM_015629.4	NM_015629.4:c.(322+1_323−1)_(697+1_698−1)del	-	-
*PRPH2*	1	HET	deletion of exon 2 in NM_000322.5	NM_000322.5:c.(581+1_582−1)_(828+1_829−1)del	-	-
*RAB28*	1	HOM	deletion of exons 5–7 in NM_001017979.3	NM_004249.3:c.(391+1_392−1)_(*1_?)del	-	-
*RP2*	1	HET	deletion of all exons in NM_006915.3	NM_006915.3:c.(?_−1)_(*1_?)del	-	-
*RP2*	1	HEMI	deletion of exon 3 in NM_006915.3	NM_006915.3:c.(768+1_769−1)_(883+1_884−1)del	-	-
*RP2*	1	HEMI	deletion of exons 2–3 in NM_006915.3	NM_006915.3:c.(102+1_103−1)_(883+1_884−1)del	-	-
*RPGR*	1	HET	deletion of exon 15 (ORF15) in NM_001034853.2	NM_001034853.2:c.(1753+1_1754−1)_(*1_?)del	-	-
*RPGRIP1*	1	HET	deletion of exons 2–3 in NM_020366.4	NM_020366.4:c.(?_−1)_(218+1_219−1)del	-	-
*TRPM1*	1	HET	deletion of exons 2–8 in NM_001252024.2	NM_001252024.2:c.(−84+1_−83−1)_(965+1_966−1)del	0.0003688 (DEL_15_146513)	-
*USH2A*	14	HET (12 cases)/ HOM (2 cases)	deletion of exons 22–24 in NM_206933.4	NM_206933.4:c.(4627+1_4628−1)_(4987+1_4988−1)del	-	179215 (Pathogenic)
*USH2A*	5	HET	deletion of exons 10–11 in NM_206933.4	NM_206933.4:c.(1644+1_1645−1)_(1971+1_1972−1)del	-	503554 (Likely pathogenic)
*USH2A*	1	HET	deletion of exons 5–37 in NM_206933.4	NM_206933.4:c.(784+1_785−1)_(7120+1_7121−1)del	-	-
*USH2A*	1	HET	deletion of exons 9–11 in NM_206933.4	NM_206933.4:c.(1550+1_1551−1)_(1971+1_1972−1)del	-	-
*USH2A*	1	HET	deletion of exons 17–24 in NM_206933.4	NM_206933.4:c.(3316+1_3317−1)_(4987+1_4988−1)del	-	-
*USH2A*	1	HET	deletion of exons 22–32 in NM_206933.4	NM_206933.4:c.(4627+1_4628−1)_(6325+1_6326−1)del	-	-

## Data Availability

All necessary data are included in the Supplementary Materials. Please contact the corresponding author to discuss the possibility of obtaining more detailed information.

## Supplementary Materials

The following supporting information can be downloaded at: https://www.mdpi.com/article/10.3390/biomedicines12061355/s1, File S1: Lists of genes covered by the subsequent panel versions and calculation of the ABCA4 haplotype frequency in causal variants with a comparison to previous data for the Polish population (Ścieżyńska et al. 2016 [53]); Figure S1: Gender distribution by initial phenotypic group for the described cohort; Figure S2: Age distribution by initial phenotypic group for the described cohort; Table S1: Gender and age information for the described cohort, including stratification into initial phenotypic groups; Table S2: Confirmatory variants for 646 solved patients; Table S3: Variants of uncertain significance for 15 likely solved patients; Table S4: List of 91 novel causative variants found in solved cases.
