# Supplementary material for: Optimised, Broad NGS Panel for Inherited Eye Diseases to Diagnose 1000 Patients in Poland
Source: Biomedicines. 2024 Jun 18;12(6):1355. doi: 10.3390/biomedicines12061355 (PMC11202224; doi:10.3390/biomedicines12061355)
Supplement: Supplementary file 1 [file biomedicines-12-01355-s001.zip › File_S1_gene_lists_and_ABCA4_haplotype_frequency.pdf]

Supplementary information for article:

Optimised, Broad NGS Panel for Inherited Eye Diseases to Diagnose 1000 Patients in Poland

1) Gene lists for three subsequent NGS panel versions:
   a) Gene list for the initial version of the panel:

HGNC ID	Gene Symbol	RefSeq ID
34	*ABCA4*	NM_000350.3
57	*ABCC6*	NM_001171.6
15868	*ABHD12*	NM_001042472.3
23338	*ACBD5*	NM_145698.5
118	*ACO2*	NM_001098.3
216	*ADAM9*	NM_003816.3
17110	*ADAMTS18*	NM_199355.4
13839	*ADGRA3*	NM_145290.4
17416	*ADGRV1*	NM_032119.4
24040	*ADIPOR1*	NM_015999.6
315	*AFG3L2*	NM_006796.3
26147	*AGBL5*	NM_021831.6
21575	*AHI1*	NM_001134831.2
348	*AHR*	NM_001621.5
359	*AIPL1*	NM_014336.5
428	*ALMS1*	NM_001378454.1
17090	*ARHGEF18*	NM_001367823.1
17146	*ARL2BP*	NM_012106.4
694	*ARL3*	NM_004311.4
13210	*ARL6*	NM_001278293.3
32685	*ARMS2*	NM_001099667.3
24102	*ARSG*	NM_001267727.2
16448	*ASRGL1*	NM_001083926.2
791	*ATF6*	NM_007348.4
10560	*ATXN7*	NM_001377405.1
28093	*BBIP1*	NM_001195305.3
966	*BBS1*	NM_024649.5
26291	*BBS10*	NM_024685.4
26648	*BBS12*	NM_152618.3
967	*BBS2*	NM_031885.5
969	*BBS4*	NM_033028.5
970	*BBS5*	NM_152384.3
18758	*BBS7*	NM_176824.3
30000	*BBS9*	NM_198428.3
12703	*BEST1*	NM_004183.4
14344	*C1QTNF5*	NM_001278431.2
1248	*C2*	NM_000063.6

1318	*C3*	NM_000064.4
1375	*CA4*	NM_000717.5
1386	*CABP4*	NM_145200.5
1393	*CACNA1F*	NM_001256789.3
20202	*CACNA2D4*	NM_172364.5
1482	*CAPN5*	NM_004055.5
29253	*CC2D2A*	NM_001378615.1
1615	*CCT2*	NM_006431.3
13733	*CDH23*	NM_022124.6
1762	*CDH3*	NM_001793.6
14550	*CDHR1*	NM_033100.4
29182	*CEP164*	NM_014956.5
28209	*CEP19*	NM_032898.5
1859	*CEP250*	NM_007186.6
29021	*CEP290*	NM_025114.4
25740	*CEP78*	NM_001330691.3
21699	*CERKL*	NM_201548.5
1260	*CFAP410*	NM_004928.3
27232	*CFAP418*	NM_177965.4
1037	*CFB*	NM_001710.6
4883	*CFH*	NM_000186.4
1940	*CHM*	NM_000390.4
24579	*CIB2*	NM_006383.4
29675	*CLCC1*	NM_001377458.1
2074	*CLN3*	NM_001042432.2
12605	*CLRN1*	NM_174878.3
19009	*CLUAP1*	NM_015041.3
2148	*CNGA1*	NM_001379270.1
2150	*CNGA3*	NM_001298.3
2151	*CNGB1*	NM_001297.5
2153	*CNGB3*	NM_019098.5
105	*CNNM4*	NM_020184.4
2186	*COL11A1*	NM_001854.4
2200	*COL2A1*	NM_001844.5
2217	*COL9A1*	NM_001851.6
2343	*CRB1*	NM_201253.3
2383	*CRX*	NM_000554.6
26193	*CSPP1*	NM_001382391.1
2509	*CTNNA1*	NM_001903.5
23198	*CYP4V2*	NM_207352.4
20603	*DHDDS*	NM_205861.3
17211	*DHX38*	NM_014003.4
2928	*DMD*	NM_004006.3
28769	*DRAM2*	NM_001349884.2
37261	*DTHD1*	NM_001170700.3
3218	*EFEMP1*	NM_001039348.3
14418	*ELOVL1*	NM_022821.4
14415	*ELOVL4*	NM_022726.4
28957	*EMC1*	NM_015047.3
3438	*ERCC6*	NM_000124.4

13281	*ESPN*	NM_031475.3
17097	*EXOSC2*	NM_014285.7
21555	*EYS*	NM_001142800.2
25808	*FAM161A*	NM_001201543.2
3602	*FBLN5*	NM_006329.4
24682	*FLVCR1*	NM_014053.4
3960	*FSCN2*	NM_012418.4
4042	*FZD4*	NM_012193.4
4221	*GDF6*	NM_001001557.4
4393	*GNAT1*	NM_144499.3
4394	*GNAT2*	NM_001377295.2
4400	*GNB3*	NM_002075.4
23026	*GNPTG*	NM_032520.5
31371	*GPR179*	NM_001004334.4
10013	*GRK1*	NM_002929.3
4598	*GRM6*	NM_000843.4
4678	*GUCA1A*	NM_001384910.1
4679	*GUCA1B*	NM_002098.6
4689	*GUCY2D*	NM_000180.4
4816	*HARS1*	NM_002109.6
26527	*HGSNAT*	NM_152419.3
4922	*HK1*	NM_000188.3
19194	*HMCN1*	NM_031935.3
5017	*HMX1*	NM_018942.3
9476	*HTRA1*	NM_002775.5
5385	*IDH3B*	NM_006899.5
29077	*IFT140*	NM_014714.4
30391	*IFT172*	NM_015662.3
18626	*IFT27*	NM_001177701.3
14313	*IFT81*	NM_014055.4
6052	*IMPDH1*	NM_000883.4
6055	*IMPG1*	NM_001563.4
18362	*IMPG2*	NM_016247.4
21474	*INPP5E*	NM_019892.6
17870	*INVS*	NM_014425.5
28949	*IQCB1*	NM_001023570.4
6174	*ITM2B*	NM_021999.5
6188	*JAG1*	NM_000214.3
6259	*KCNJ13*	NM_002242.4
19698	*KCNV2*	NM_133497.4
22219	*KIAA1549*	NM_001164665.2
6388	*KIF11*	NM_004523.4
15865	*KIZ*	NM_018474.6
15646	*KLHL7*	NM_001031710.3
6481	*LAMA1*	NM_005559.4
31923	*LCA5*	NM_001122769.3
6685	*LRAT*	NM_004744.5
24783	*LRIT3*	NM_198506.5
6697	*LRP5*	NM_002335.4
6741	*LZTFL1*	NM_020347.4

6816	*MAK*	NM_001242957.3
6888	*MAPKAPK3*	NM_001243925.2
7027	*MERTK*	NM_006343.3
16877	*MFN2*	NM_014874.4
18121	*MFRP*	NM_031433.4
28486	*MFSD8*	NM_001371596.2
31582	*MIR204*	NR_029621.1
7108	*MKKS*	NM_170784.3
7121	*MKS1*	NM_017777.4
26784	*MTRFR*	NM_152269.5
7467	*MTTP*	NM_001386140.1
7530	*MVK*	NM_000431.4
7606	*MYO7A*	NM_000260.4
15625	*NBAS*	NM_015909.4
7678	*NDP*	NM_000266.4
7745	*NEK2*	NM_002497.4
7762	*NEUROD1*	NM_002500.5
17877	*NMNAT1*	NM_022787.4
7905	*NPHP1*	NM_001128178.3
7907	*NPHP3*	NM_153240.5
19104	*NPHP4*	NM_015102.5
7974	*NR2E3*	NM_014249.4
7975	*NR2F1*	NM_005654.6
8002	*NRL*	NM_001354768.3
8082	*NYX*	NM_001378477.3
8091	*OAT*	NM_000274.4
2567	*OFD1*	NM_003611.3
8140	*OPA1*	NM_130837.3
8142	*OPA3*	NM_025136.4
1012	*OPN1SW*	NM_001385125.1
8522	*OTX2*	NM_021728.4
15894	*PANK2*	NM_001386393.1
8616	*PAX2*	NM_000278.5
34383	*PCARE*	NM_001029883.3
14674	*PCDH15*	NM_001384140.1
8754	*PCYT1A*	NM_001312673.2
8785	*PDE6A*	NM_000440.3
8786	*PDE6B*	NM_000283.4
8787	*PDE6C*	NM_006204.4
8789	*PDE6G*	NM_002602.4
8790	*PDE6H*	NM_006205.3
26257	*PDZD7*	NM_001195263.2
8850	*PEX1*	NM_000466.3
9717	*PEX2*	NM_000318.3
8860	*PEX7*	NM_000288.4
8896	*PGK1*	NM_000291.4
8940	*PHYH*	NM_006214.4
21043	*PITPNM3*	NM_031220.4
9038	*PLA2G5*	NM_000929.3
11397	*PLK4*	NM_014264.5

16268	*PNPLA6*	NM_001166114.2
30836	*POC1B*	NM_172240.3
26658	*POC5*	NM_001099271.2
19139	*POMGNT1*	NM_017739.4
32528	*PRCD*	NM_001077620.3
13998	*PRDM13*	NM_021620.4
9402	*PRKCG*	NM_002739.5
9454	*PROM1*	NM_006017.3
17348	*PRPF3*	NM_004698.4
15446	*PRPF31*	NM_015629.4
17349	*PRPF4*	NM_001244926.2
15860	*PRPF6*	NM_012469.4
17340	*PRPF8*	NM_006445.4
9942	*PRPH2*	NM_000322.5
9462	*PRPS1*	NM_002764.4
9768	*RAB28*	NM_001017979.3
18286	*RAX2*	NM_001319074.4
9884	*RB1*	NM_000321.3
9921	*RBP3*	NM_002900.3
9922	*RBP4*	NM_006744.4
18243	*RCBTB1*	NM_018191.4
19689	*RD3*	NM_001164688.2
17964	*RDH11*	NM_016026.4
19977	*RDH12*	NM_152443.3
9940	*RDH5*	NM_002905.5
30078	*REEP6*	NM_138393.4
9990	*RGR*	NM_001012720.2
10004	*RGS9*	NM_003835.4
30304	*RGS9BP*	NM_207391.3
10012	*RHO*	NM_000539.3
17282	*RIMS1*	NM_014989.7
10024	*RLBP1*	NM_000326.5
10254	*ROM1*	NM_000327.4
10263	*RP1*	NM_006269.2
15946	*RP1L1*	NM_178857.6
10274	*RP2*	NM_006915.3
10288	*RP9*	NM_203288.2
10294	*RPE65*	NM_000329.3
10295	*RPGR*	NM_001034853.2
13436	*RPGRIP1*	NM_020366.4
29168	*RPGRIP1L*	NM_015272.5
10457	*RS1*	NM_000330.4
18647	*RTN4IP1*	NM_032730.5
10521	*SAG*	NM_000541.5
28706	*SAMD11*	NM_001385641.1
10671	*SDCCAG8*	NM_006642.5
10729	*SEMA4A*	NM_022367.4
10975	*SLC24A1*	NM_004727.3
25198	*SLC25A46*	NM_138773.4
29326	*SLC7A14*	NM_020949.3

30859	*SNRNP200*	NM_014014.5
20423	*SPATA7*	NM_018418.5
11256	*SPP2*	NM_006944.3
11714	*TEAD1*	NM_021961.6
11817	*TIMM8A*	NM_004085.4
11822	*TIMP3*	NM_000362.5
11849	*TLR3*	NM_003265.3
11850	*TLR4*	NM_138554.5
25382	*TMEM126A*	NM_032273.4
25018	*TMEM216*	NM_001173990.3
14432	*TMEM237*	NM_001044385.3
21653	*TOPORS*	NM_005802.5
12269	*TREX1*	NM_033629.6
16380	*TRIM32*	NM_012210.4
17341	*TRNT1*	NM_182916.3
7146	*TRPM1*	NM_001252024.2
21641	*TSPAN12*	NM_012338.4
20087	*TTC8*	NM_144596.4
19963	*TTLL5*	NM_015072.5
12404	*TTPA*	NM_000370.3
12406	*TUB*	NM_177972.3
16691	*TUBGCP4*	NM_014444.5
18127	*TUBGCP6*	NM_020461.4
12423	*TULP1*	NM_003322.6
12565	*UNC119*	NM_005148.4
12597	*USH1C*	NM_153676.4
16356	*USH1G*	NM_173477.5
12601	*USH2A*	NM_206933.4
2464	*VCAN*	NM_004385.5
2183	*VPS13B*	NM_152564.5
28027	*WDPCP*	NM_015910.7
18340	*WDR19*	NM_025132.4
12762	*WFS1*	NM_006005.3
16361	*WHRN*	NM_015404.4
20041	*ZNF408*	NM_024741.3
16762	*ZNF423*	NM_001379286.1
26498	*ZNF513*	NM_144631.6

b) Genes added incrementally to the second panel version:

**HGNC ID**	**Gene symbol**	**RefSeq ID**
21014	*ANTXR1*	NM_032208.3
25419	*ARL13B*	NM_001174150.2
20730	*ARMC9*	NM_001352754.2
801	*ATP1A3*	NM_152296.5
24123	*B9D1*	NM_015681.6
28636	*B9D2*	NM_030578.4
24564	*C2CD3*	NM_001286577.2

28163	*CCDC28B*	NM_024296.5
3231	*CELSR2*	NM_001408.3
24866	*CEP104*	NM_014704.4
26690	*CEP120*	NM_001375405.1
12370	*CEP41*	NM_018718.3
24212	*CISD2*	NM_001008388.5
25801	*CPLANE1*	NM_001384732.1
10664	*CWC27*	NM_005869.4
16410	*DNAJC30*	NM_032317.3
2973	*DNM1L*	NM_012062.5
2962	*DYNC2H1*	NM_001377.3
28296	*DYNC2I2*	NM_052844.4
3360	*ENSA*	NM_004436.4
24659	*EXOC8*	NM_175876.5
29162	*FAM149B1*	NM_173348.2
3642	*FDXR*	NM_024417.5
8079	*FRMD7*	NM_194277.3
4392	*GNAS*	NM_000516.7
20145	*GPR143*	NM_000273.3
26558	*HYLS1*	NM_001134793.2
21424	*IFT74*	NM_025103.4
29068	*KATNIP*	NM_015202.5
19960	*KIAA0586*	NM_001329943.3
29110	*KIAA0753*	NM_014804.3
6320	*KIF3B*	NM_004798.4
30497	*KIF7*	NM_198525.3
29622	*MCAT*	NM_173467.5
25979	*MIEF1*	NM_019008.6
9936	*OPN1LW*	NM_020061.6
4206	*OPN1MW*	NM_000513.2
8788	*PDE6D*	NM_002601.4
8819	*PDXK*	NM_003681.5
18145	*PHF6*	NM_001015877.2
23352	*PIBF1*	NM_006346.4
9325	*PPT1*	NM_000310.4
9456	*PROS1*	NM_000313.4
10680	*SDHA*	NM_004168.4
11033	*SLC4A7*	NM_001321103.2
11237	*SPG7*	NM_003119.4
11317	*SSBP1*	NM_003143.3
16466	*SUFU*	NM_016169.4
26113	*TCTN1*	NM_001082538.3
25774	*TCTN2*	NM_024809.5
24519	*TCTN3*	NM_015631.6
28128	*TMEM107*	NM_183065.4
26944	*TMEM138*	NM_016464.5
37234	*TMEM231*	NM_001077418.3
28396	*TMEM67*	NM_153704.6
17861	*TRAF3IP1*	NM_015650.4
25660	*TTC21B*	NM_024753.5

12843	*YME1L1*	NM_014263.4
12309	*ZNHIT3*	NM_004773.4

c) Genes added incrementally to the third panel version:

**HGNC ID**	**Gene symbol**	**RefSeq ID**
360	*AIRE*	NM_000383.4
403	*ALDH3A2*	NM_000382.3
20917	*ALPK1*	NM_025144.4
451	*AMACR*	NM_014324.6
11891	*CLEC3B*	NM_003278.3
2076	*CLN5*	NM_006493.4
2077	*CLN6*	NM_017882.3
2079	*CLN8*	NM_018941.4
26169	*CTC1*	NM_025099.6
2514	*CTNNB1*	NM_001904.4
2529	*CTSD*	NM_001909.5
3439	*ERCC8*	NM_000082.4
3604	*FBN2*	NM_001999.4
20883	*GDPD1*	NM_182569.4
4601	*GRN*	NM_002087.4
23302	*HKDC1*	NM_025130.4
5384	*IDH3A*	NM_005530.3
29669	*IFT43*	NM_001102564.3
5961	*IKBKG*	NM_001099857.5
6694	*LRP2*	NM_004525.3
11957	*MED12*	NM_005120.3
24858	*MFF*	NM_001277062.2
24525	*MMACHC*	NM_015506.3
29678	*MSTO1*	NM_018116.4
7711	*NDUFS4*	NM_002495.4
8859	*PEX6*	NM_000287.4
8999	*PISD*	NM_001326411.2
9399	*PRKCD*	NM_006254.4
17283	*RIMS2*	NM_001348484.3
17296	*RRM2B*	NM_015713.5
13081	*SCAPER*	NM_020843.4
10892	*SIX6*	NM_007374.3
11052	*SLC6A6*	NM_003043.6
17884	*SPATA3*	NM_139073.5
25812	*SRD5A3*	NM_024592.5
11824	*TINF2*	NM_001099274.3
25295	*TLCD3B*	NM_031478.6
27344	*TMEM218*	NM_001258244.2
2073	*TPP1*	NM_000391.4
20771	*TUBB4B*	NM_006088.6
20080	*USP45*	NM_001346022.3
2183	*VPS13B*	NM_152564.5

18326	*YPEL2*	NM_001005404.4
20761	*ZFYVE26*	NM_015346.4

2) Calculation of the *ABCA4* haplotype frequency in causal variants with a comparison to previous data for the Polish population Ścieżyńska et al. 2016 [53]:

   To compare the *ABCA4* complex haplotype p.(Leu541Pro;Ala1038Val) with the previous work [53], we restricted our set of positive samples to those with confirmed variants identified in the *ABCA4* gene, as the previous work only considered molecular findings in the *ABCA4* gene.

   We calculated the frequency of the haplotype p.(Leu541Pro;Ala1038Val) in all positive patients from Supplementary table 2 of the Ścieżyńska et al. 2016 [53]. In 65 positive samples the haplotype p.(Leu541Pro;Ala1038Val) was identified 6 times as homozygote and 24 times as heterozygote. This gives a total of 36 alleles of the haplotype p.(Leu541Pro;Ala1038Val) for 130 alleles, which estimates the frequency of the haplotype p.(Leu541Pro;Ala1038Val) in positive samples to be 27.7%.
   Subsequently, among 135 positive samples in our cohort with confirmatory variants in *ABCA4* gene, we found 12 cases with the haplotype p.(Leu541Pro;Ala1038Val) as homozygote and 59 cases with heterozygous haplotype p.(Leu541Pro;Ala1038Val) in compound heterozygote with other causal *ABCA4* variant. These findings led to haplotype p.(Leu541Pro;Ala1038Val) frequency estimation of about 30.7% (83 alleles with haplotype divaded by 270 alleles in total). The difference in frequency between present study and the previous work of Ścieżyńska is not significant (two-tailed Fisher's exact probability test, p>0.05).
