# Supplementary figures and images for: Optimised, Broad NGS Panel for Inherited Eye Diseases to Diagnose 1000 Patients in Poland
Source: Biomedicines. 2024 Jun 18;12(6):1355. doi: 10.3390/biomedicines12061355 (PMC11202224; doi:10.3390/biomedicines12061355)

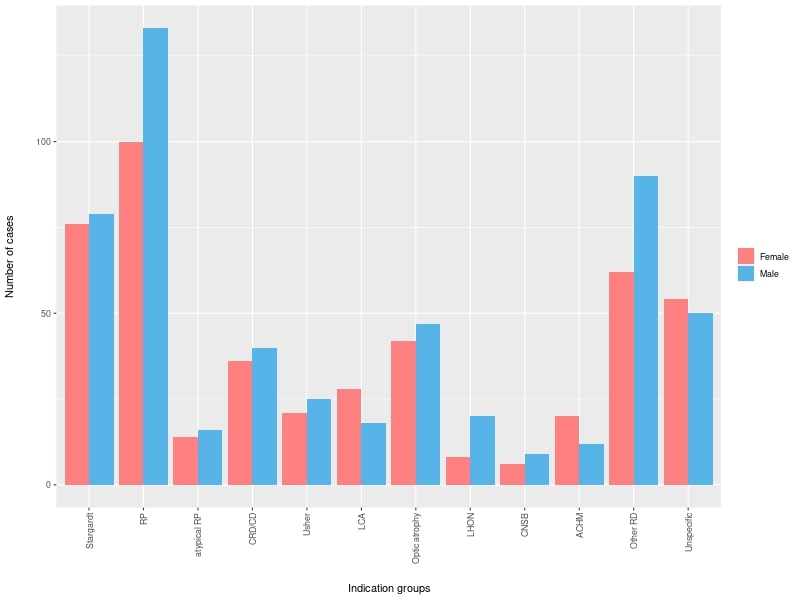

Supplement: Supplementary file 1 [file biomedicines-12-01355-s001.zip › Supplementary_Fig_1_Females_and_males_per_indication_group.jpg]

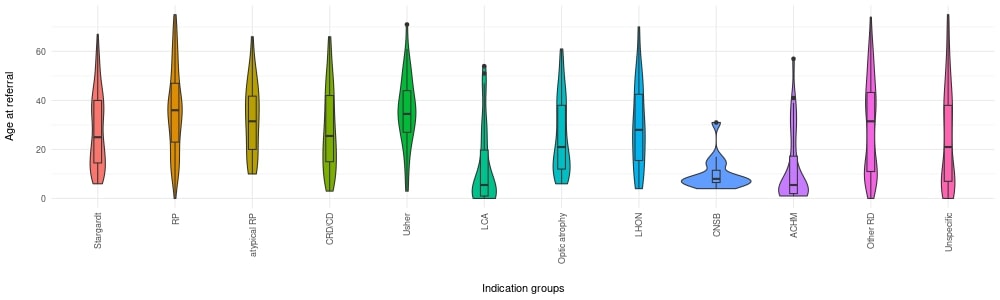

Supplement: Supplementary file 1 [file biomedicines-12-01355-s001.zip › Supplementary_Fig_2_Age_at_refferal_per_indication_group_violin_plot.jpg]
